# An HTA systems decision-support toolbox for short and long-term healthcare and economic perspectives in an Italian hospital

**DOI:** 10.1017/S0266462325103176

**Published:** 2025-10-16

**Authors:** Fabiano Bini, Alessia Finti, Michela Franzò, Flavia Grianti, Carmen D’Anna, Stefano Lazzari, Franco Marinozzi

**Affiliations:** 1 Department of Mechanical and Aerospace Engineering, Faculty of Civil and Industrial Engineering, University of Rome La Sapienza, Rome, Italy; 2 Department of Medico-Surgical Sciences and Biotechnologies, Faculty of Pharmacy and Medicine, University of Rome La Sapienza, Rome, Italy; 3 UOSD Ingegneria Clinica, San Giovanni-Addolorata Hospital, Rome, Italy

**Keywords:** costs and cost analysis, technology assessment, biomedical, analytic hierarchy process (AHP), health technology assessment (HTA)

## Abstract

**Objectives:**

The introduction of a new healthcare technology within the technological facilities of a hospital is a complex action that must go through the mandatory decision-making process of health technology assessment (HTA). Nowadays, developing a universal HTA model poses a significant challenge within the current landscape. This paper describes the proposal of a novel supporting healthcare technology evaluation toolbox, aligned with the principles of the European Network for Health Technology Assessment (EUnetHTA) shared by the Regulation (EU) 2021/2282 on Health Technology Assessment (HTAR).

**Methods:**

The proposed toolbox relies on a MATLAB-based multicriteria algorithm that mirrors the evaluative procedure following the hierarchical framework of the analytic hierarchy process. The evaluation framework involves clinical and non-clinical aspects leading to the choice of the best alternative, among the evaluated technologies, to be introduced in the technological infrastructure of the hospital. Moreover, the toolbox incorporates robust economic analysis capabilities, crucial for determining the requisite number of annual hospital procedures to ensure economic equilibrium and mitigate financial risks. Additionally, it computes the payback period, essential for evaluating the economic feasibility of technology investments. HTA evaluations at San Giovanni Addolorata Hospital demonstrate its application.

**Results:**

The toolbox exemplifies its efficacy in supporting informed decision-making processes, regarding the adoption of technologies like robotic systems for neurosurgery and angiographic systems, in terms of economic sustainability and clinical effectiveness.

**Conclusions:**

This study underscores the toolbox’s role in advancing HTA methodologies and enhancing the efficiency and sustainability of healthcare technology integration.

## Introduction

Italy’s National Health Service (SSN) provides universal, publicly funded healthcare, organized regionally with a mix of public and private providers (1). SSN faces the issues of resource allocation, cost containment, and diffusion of technology, requiring effective decision-making mechanisms at the hospital level. Health technology assessment (HTA) evaluates the clinical, economic, social, and ethical implications of healthcare technologies, supporting decision-making for a fair and efficient healthcare system (2). It is mandatory in Europe according to Directive 2011/24/EU (3), which promoted Member States’ cooperation in HTA through the European Network for Health Technology Assessment (EUnetHTA) (4;5), a collaborative initiative for sustainable HTA practices. Furthermore, Regulation (EU) 2021/2282 on HTA (HTAR) defines the modalities of cooperation among EU Member States in HTA by joint clinical assessments and voluntary cooperation (6).

Within this regulatory context, some HTA methodologies are commonly adopted in Europe. EUnetHTA’s HTA Core Model is a notable methodological framework guiding HTA reports and rapid effectiveness assessment (REA) reports (7). Core model structures HTA across nine domains, including technical, clinical, economic, organizational, and ethical aspects (7): health problem and current use of technology, description and technical characteristics of technology, safety, clinical effectiveness, costs and economic evaluation, ethical analysis, organizational, social, and legal aspects. A 2018 European Commission mapping of HTA methodologies across EU countries and Norway revealed key differences in centralized (e.g., the UK’s NICE) versus decentralized approaches, levels of stakeholder involvement and evaluation criteria. Common methodologies include clinical effectiveness evaluation through systematic reviews and meta-analyses, and economic evaluations such as cost-effectiveness analysis, cost-utility analysis, and cost–benefit analysis. In addition, HTA also includes the assessment of social and ethical impacts (8).

While traditional HTA focuses on national-level decisions, hospital-based HTA (HB-HTA) evaluates technologies within the hospital setting by focusing on their clinical, economic, and operational impacts (9). Among existing decision-making strategies in HB-HTA, the AdHopHTA (adopting hospital-based health technology assessment) (10) provided standardized tools for supporting the evaluation of healthcare technologies at the hospital level. These tools help generate full HTA reports regarding clinical, economic, and organizational aspects. In contrast, rapid HTA reports (11;12) provide clinical and economic evaluation, excluding social and organizational aspects, delivering timely evidence to decision-makers facing resource and time limitations.

This paper proposes a unique supporting toolbox for rapid HB-HTA evaluations within an Italian hospital, aiming at support decision-making in a fast and comprehensive way. Although validated on large medical devices, the modular structure is extendable in the future to pharmaceuticals. Developed by the HTA team at San Giovanni Addolorata Hospital in Rome, it responds to the institutional need for rapid, multidisciplinary assessments that integrate both clinical and economic aspects, unlike traditional REAs (13), which are often limited to clinical aspects. The toolbox has been applied to support HB-HTA evaluations for large healthcare equipment assessment at the Clinical Engineering Department of San Giovanni Addolorata Hospital. Aligned with the principles of EUnetHTA (4) and the current directive, it has been applied to draft rapid HTA reports. Designed to answer the hospital’s need of an efficient solution for rapid multidisciplinary assessments, the toolbox reflects the EUnetHTA model, dividing healthcare technology analysis into clinical and non-clinical domains. It aligns with EUnetHTA’s core model and rapid HTA reports, covering clinical (health issues, technical characteristics, safety, effectiveness) and non-clinical (economic) domains (7). Additionally, given the large potential of multicriteria decision analysis (MCDA) methods in supporting HTA agencies in setting healthcare priorities (14), the toolbox is based on a MATLAB multicriteria algorithm reflecting the evaluative process based on the hierarchical structure of the analytic hierarchy process (AHP) (15). The AHP method has previously been integrated into healthcare decision systems for hospital settings (16) leading to a facilitation of the prioritization of the evaluation criteria. Furthermore, MCDA methods have been proposed as a possible solution to critical issues for medical devices evaluation (17).

Given the significant role of biomedical technologies in increasing healthcare costs (18), despite conferring significant health benefits, their economic evaluation has previously been integrated within comprehensive frameworks such as the HTA core model. Furthermore, national economic approaches may be misaligned with hospital decision-making since they focus on macroeconomic aspects (19). The adoption of HB-HTA addresses this gap by analyzing the costs incurred by the hospital’s HTA team and the economic-financial balance (9). To support economic evaluations within the toolbox, capital budgeting techniques are integrated to capture both short- and long-term financial implications of healthcare investments (20). There are several evaluation methods relying on these concepts (21). The main methodologies based on cash flows are net present value, internal rate of return, and payback period (PBP). Additionally, analytical accounting is also used, especially in the cost-volume-profit (CVP) model, a directional control tool supporting management across economic alternatives. CVP model, or break-even analysis, defines the degree of enterprise elasticity in relation to cost structure. The outcome is the break-even point (BEP), representing economic equilibrium (22;23). These tools are essential for developing a universal HB-HTA model in a fragmented European HTA context.

The toolbox combines economic and multidisciplinary (i.e., technical, clinical, and safety) evaluations, analyzing investments in the short and long-term, through key indicators which HTA team must consider while evaluating the feasibility of the investments within their limited budget: the number of annual hospital performances required to maintain economic equilibrium and avoid loss, and the number of years required to recover the investment expenditure. Finally, it enables the execution of a retrospective analysis to identify the most significant economic indicators.

Specifically, the application was involved in the HTA evaluation concerning the acquisition of an angiographic system for constructing a hybrid operating room and a robotic system for neurosurgery at San Giovanni Addolorata Hospital.

The study aims to optimize the healthcare decisions through a multidisciplinary HTA toolbox. In the short term (1 year), it seeks to streamline decision-making by providing fast, consistent evaluations for adopting high-technology systems such as imaging and robotic surgery ones. Over the long term (up to 8 years), it aims to improve hospital economic efficiency by optimizing resource management and increasing the number of services provided annually.

## Materials and methods

### Multicriterial decision analysis for HTA

After reviewing existing methodologies, the healthcare technology evaluation support toolbox was built on an MCDA algorithm. The AHP method was chosen because of its proven applicability in HTA evaluations as demonstrated by Ritrovato et al. (16). MCDA methods handle the multidisciplinarity of the HTA process, enabling decision-makers to compare and weigh different evaluation criteria through a hierarchical model. The decision-making process is automated in the MATLAB (v. R2024a) environment using an interface based on a sentient algorithm that mirrored the AHP method (15) for MCDA.

The HTA team involved in rapid report assessments was composed of multidisciplinary personnel, including clinical engineers and medical, legal, and administrative staff. The core technical team had a senior manager (>20 years’ experience) and two clinical engineers (>10 years’ experience). While the AHP model was implemented by the technical team, the domain and sub-domain weighting was determined together with a balanced sample of medical, technical, and administrative stakeholders. The MCDA process was supported by the DIMA Bioengineering Research Group of Sapienza University of Rome, with over 20 years of experience in decision analysis. The HTA team’s qualitative parameters were translated into quantitative input parameters according to Saaty’s Scale (15), which assigns numerical values (1–9) to pairwise comparisons, where 1 indicates equal importance and 9 denotes extreme importance. The algorithm then translated the input parameters into a numerical model driving the entire evaluation process. Once the clinical question (GOAL: evaluation of a specific healthcare technology) was identified, the team used an interactive interface for defining and weighting investigation domains. These domains were divided into *n*-criteria at the first level (technical characteristics, clinical effectiveness, safety, economic, and market analysis), each branching into *m*-criteria at the second level. The criteria aligned with those shared by the HTA procedures manual from AGENAS (The Italian National Agency for Regional Healthcare Services) (11), which is informed by European guidelines from EUnetHTA for developing a rapid HTA report and is currently being updated (12). Table 1 presents the sub-criteria used for the two evaluated technologies, grouped by domain. Sub-criteria are customizable for device-specific evaluation.

**Table 1.** Investigation domains included in the toolbox during the evaluation of robots for neurosurgery and angiographs

Criteria/domain	Subcriteria	
	Robot for neurosurgery	Angiograph
Technical Specifications	Compatibility with intra-operative systems already adopted by the hospital	Rotation range of the arch in RAO (Right Anterior Oblique) - LAO (Left Anterior Oblique) head position
	Ease of use	Useful arch depth
	Consumables required for surgical interventions	Focus-detector distance (SID - Source-to-Image Distance)
	Degrees of freedom	3D acquisition rotation speed
	Navigation methods of the neuronavigational system	Pixel size
	Accuracy of the device	DQE (detective quantum efficiency)
	Surgical drill hole size	Anode cooling speed
Clinical Effectiveness	Length of hospital stay after robotic neurosurgery	Dose exposure during the use of the device
	Need for revision surgery after robotic neurosurgery	AI (artificial intelligence) software for the regulation of acquisition parameters
	Clinical care settings in which the device can be employed	Ease of access to anatomical districts
Safety	Categories of at-risk patients for robotic neurosurgery	Accessibility for sanitization
	Complication rate during robotic neurosurgery	Ease of access to anatomical districts
	Post-robotic neurosurgery complication rate	
	Radiation exposure during robotic neurosurgery	
	Stabilization systems of the device	
	Alarm systems of the device	
Economic and market analysis	Technology presence on national territory	Technology presence on national territory
	BEP (break-even point) and/or PBP (payback period)	BEP and/or PBP

*Note:* These domains are divided into four criteria at the first level (technical characteristics, clinical effectiveness, safety, economic and market analysis), each branching into m-criteria at the second level. The weights of these criteria are determined within the evaluation process through Saaty’s Scale of absolute numbers (15). The criteria in the first column align with those shared by the HTA procedures manual from AGENAS (The Italian National Agency for Regional Healthcare Services) (11), which is informed by European guidelines from EUnetHTA for developing a rapid HTA report and is currently being updated (12). The sub-criteria are technology-specific, allowing for an evaluation tailored to the device.

Before running the toolbox, the “clinical problem” (7) domain was assessed through an informational questionnaire administered to the medical department director involved in the adoption of the new technology. The questionnaire collecting data on technology-associated diseases (epidemiology, interventions, hospital stay) is reported in Appendix B, Supplementary Material.

The algorithm’s final step combined weights and performance scores of each technology, both assigned by HTA team, to compare alternatives and propose the one best meeting clinical effectiveness, economic sustainability, and technical requirements.

Regarding the economic and market analysis domain, the interface allowed to conduct a detailed economic analysis that generated two standard economic indicators for all technologies: PBP and BEP, rapidly providing both short- and long-term evaluation for economic feasibility of investments. Depending on the HTA team’s output of interest, they either selected BEP to determine the annual service volume needed to avoid loss or PBP to compute the years required to recoup the investment. The chosen indicator then became the first sub-criterion of the “Economic and Market Analysis” domain. The second sub-criterion of the domain was “presence of the evaluated technology within the national territory” which aimed to observe its diffusion and actual implementation.

Operationally, the interface followed the AHP method, where the HTA team provided judgements based on Thomas L. Saaty’s scale of absolute numbers (15). The HTA team agreed on an average absolute number for each analyzed criteria to input the algorithm. Pairwise comparisons determined weights for each criterion and sub-criterion, showing their importance in the evaluation process. Weights respected the condition of normality (i.e., they sum up to 1 (24)) and were expressed as percentages.

By following the theory of AHP (15), the algorithm checked the consistency of these judgements, and, through the construction of a judgement matrix, it proceeded with pairwise comparisons at each level of interest.

The toolbox was implemented on a carefully selected and well-balanced sample, including two distinct and heterogeneous groups: angiographic systems and robotic neurosurgical systems. All evaluation domains—technical, clinical, safety, and economic—are fully integrated into the AHP process.

### Long- and short-term predictive economic analysis

Economic evaluation within the AHP process resulted from careful analysis. Break even analysis and pay-back financial evaluation methodologies were implemented, since they provided economic indicators to support the HTA team of the hospital trust in evaluating new technologies.

Considering the division of hospital equipment categories established by EU Regulation 2017/745 into diagnostic and surgical equipment macro-categories, the correct allocation of cost items to the technology under examination based on its classification was crucial.

BEP and PBP required a cost and revenue analysis of the technology and its alternatives. Cost analysis through a direct costing approach enabled the identification of cost items attributable to equipment compatible with hospital trust’s activities. Cost items were categorized into fixed (purchase, maintenance, amortization) and variable costs (operating room, inpatient, consumables, disposables, sterilization).

In the regionalized SSN, hospital care reimbursement is based on regional tariffs (18). The toolbox included tariffs associated with specialist outpatient care services listed in Appendix 4 to the DPCM (Council of Ministry President’s Decree) of January 12, 2017 (25) and tariffs for hospital care services (diagnosis-related group [DRG] tariffs) specific to the Lazio region. Given the multitude of DRGs associated with the reviewed technology, the hospital trust was considered a multi-product company. Thus, a constant production/sales mix a priori was hypothesized. The percentages of volumes, defining the proportional weight that each type of DRG contributes to the total annual services delivered, were calculated through implemented Formulas in Appendix A, Supplementary Material. The minimum quantity of services that must be delivered at the corporate level to avoid economic loss (BEP) was identified, considering the services deliverable with the reviewed technology (Formula S1, Supplementary Material).

The weighted price was calculated (Formula S2, Supplementary Material) by summing the products of DRG-specific prices and volume percentages. Similarly, Formula S3, Supplementary Material allowed to calculate the weighted unit variable cost, by summing the products of the volume percentage and the unit variable cost associated with the i-th DRG.

The short-term annual cost-revenues analysis led to a long-term financial scrutiny for decision-making. More specifically, the short-term horizon referred to a 1-year period, the medium-term to 3–5 years, and the long-term analysis considered a timeframe up to 8 years, in line with the average expected lifespan of large-scale hospital equipment.

PBP (Formula S4, Supplementary Material) determined the *k* years required for the annual cash flow generated by the investment over time to allow the hospital trust to recover the initial expenditure incurred by introducing the new technology.

Considering an average equipment lifespan of 8 years (26), break-even years were crucial to avoid exceeding its useful life. It was essential to establish a payback years limit to define new quantities of services to be provided for each DRG, as economic analysis often requires pre-setting conditions and predictive analysis for investment feasibility.

Formula S5, Supplementary Material determined the total break-even quantity Q to be provided, while maintaining fixed the desired payback years from the investment, to avoid an economically disadvantaged situation. This enabled identification of new necessary service volumes to recover the investment in a low number of payback years.

A sensitivity analysis identified the most impactful economic indicators for planning the hospital’s economic activities and to assess their influence on economic outcomes. It was executed by varying key parameters (e.g., DRG tariffs and variable costs) by ±10 percent to assess their impact on economic outcomes.

## Results

The HTA team used the toolbox to evaluate two technologies for integration into its technological infrastructure, identified as acquisition priorities by the hospital governance: a robotic neurosurgery system and an angiographic system for a hybrid operating room. These were assessed according to EUnetHTA domains but differ in their purposes and applications. For instance, evaluating a robotic system focuses on degrees of freedom or complication rates, while an angiographic system is assessed through arc depth and anatomical access. The interface allowed the HTA team to define and weigh technology-specific evaluation parameters before comparing alternatives.

**Figure 1.**
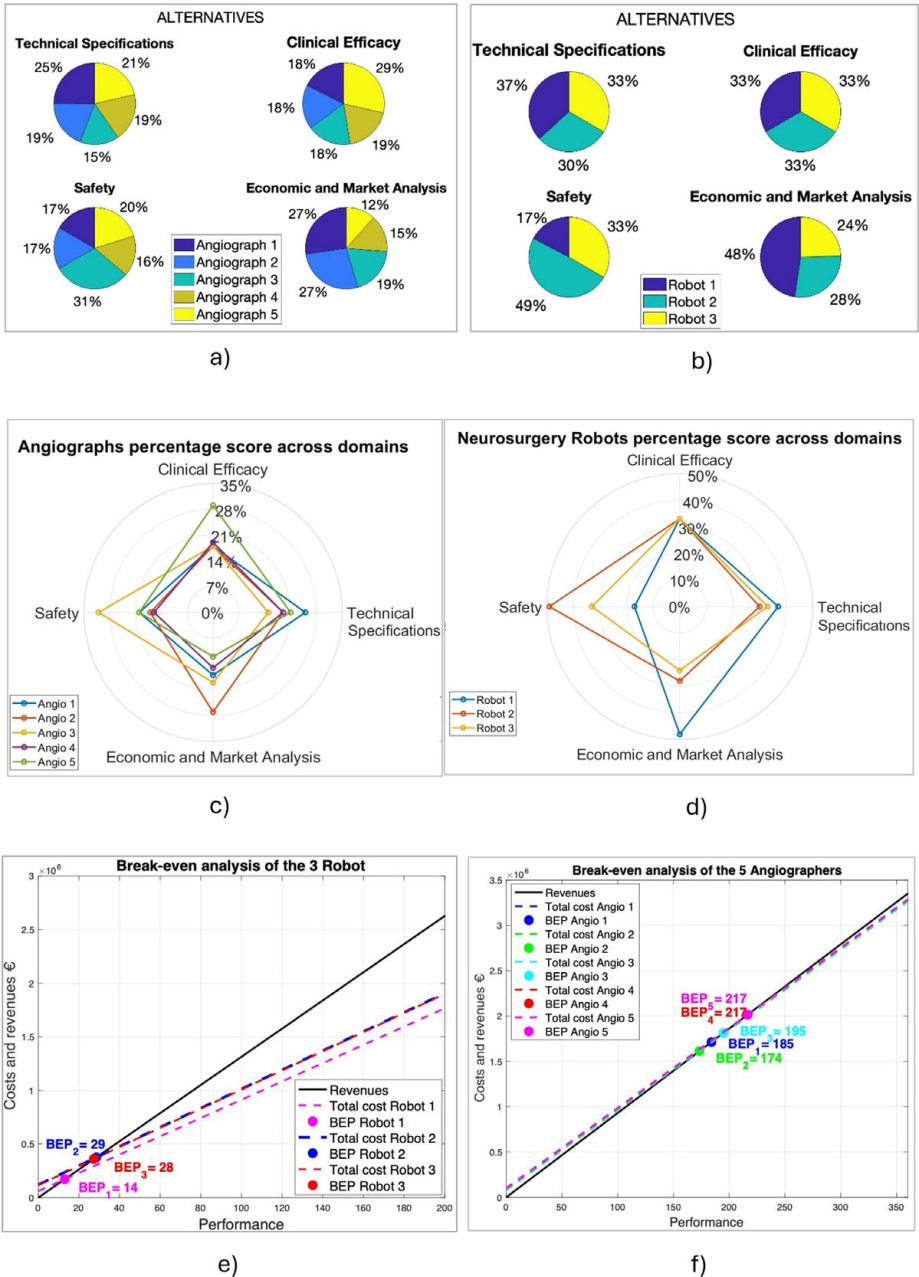
Outcome of the evaluation of the examined alternatives through the AHP toolbox. (A), (B) Results are represented through the percentage distribution of the score reached, according to each sub criteria, by the angiography systems (A) and the neurosurgery robots (B). (C), (D) Radar charts summarizing the percentage scores of each alternative in the four domains, allowing visual comparison of angiographs (C) and neurosurgery robots (D). (E), (F) Representation of the Break-Even Point expressed in minimum procedures to be delivered in order not to fall into a situation of economic loss is lower than the number of delivered annual procedures both for angiographic and robotic systems.

Figure 1A,B shows the weighted percentage contributions of each device across four evaluation domains, as computed by the AHP algorithm, while Figure 1C,D provides a radar chart overview to facilitate cross-domain comparison. Three neurosurgery robots were considered (Figure 1A). Robot 1 achieved the highest score, driven by a 37 percent contribution in technical and economic performance, despite a safety rating of 17 percent due to less advanced safety systems. Robot 2, despite a safety contribution of 33 percent of the total score, was slightly less performant (30 percent).

Robot 2 and Robot 3 scored 28 percent and 24 percent in the fourth criterion. Robot 2’s higher costs were balanced by its higher Italian market penetration. Robot 3 balanced technology and safety but had a high purchase cost.

Economic analysis results are shown in Figure 1E. The short-term analysis showed that the minimum BEP required was well below the annual performance, confirming the economic feasibility of each alternative. The minimum BEP for each robot is equal to 14 performances for Robot 1 and 28 for Robot 2 and 3, all lower than the expected number of neurosurgical procedures performed annually at the hospital.

PBP was used for comparing the alternatives, demonstrating the economic victory of Robot 1 due to its lower purchase cost while maintaining similar variable costs across the alternatives. However, the payback years for the purchase of the robot appear to be less than 2 years for each alternative.

Among the five angiographic systems (Figure 1B), Angiograph 1 is the highest scored system with a 27 percent score within the toolbox. It is driven by 25 percent in technical performance, while maintaining comparable clinical effectiveness (18 percent). However, despite a 6-year payback, its market penetration in Italy is higher.

Angiograph 2 excels in economic and market analysis, with a 5-year payback. However, it does not perform as well in other sub-criteria. Angiograph 3 shows decent performance across all aspects and excels in safety (31 percent).

**Figure 2.**
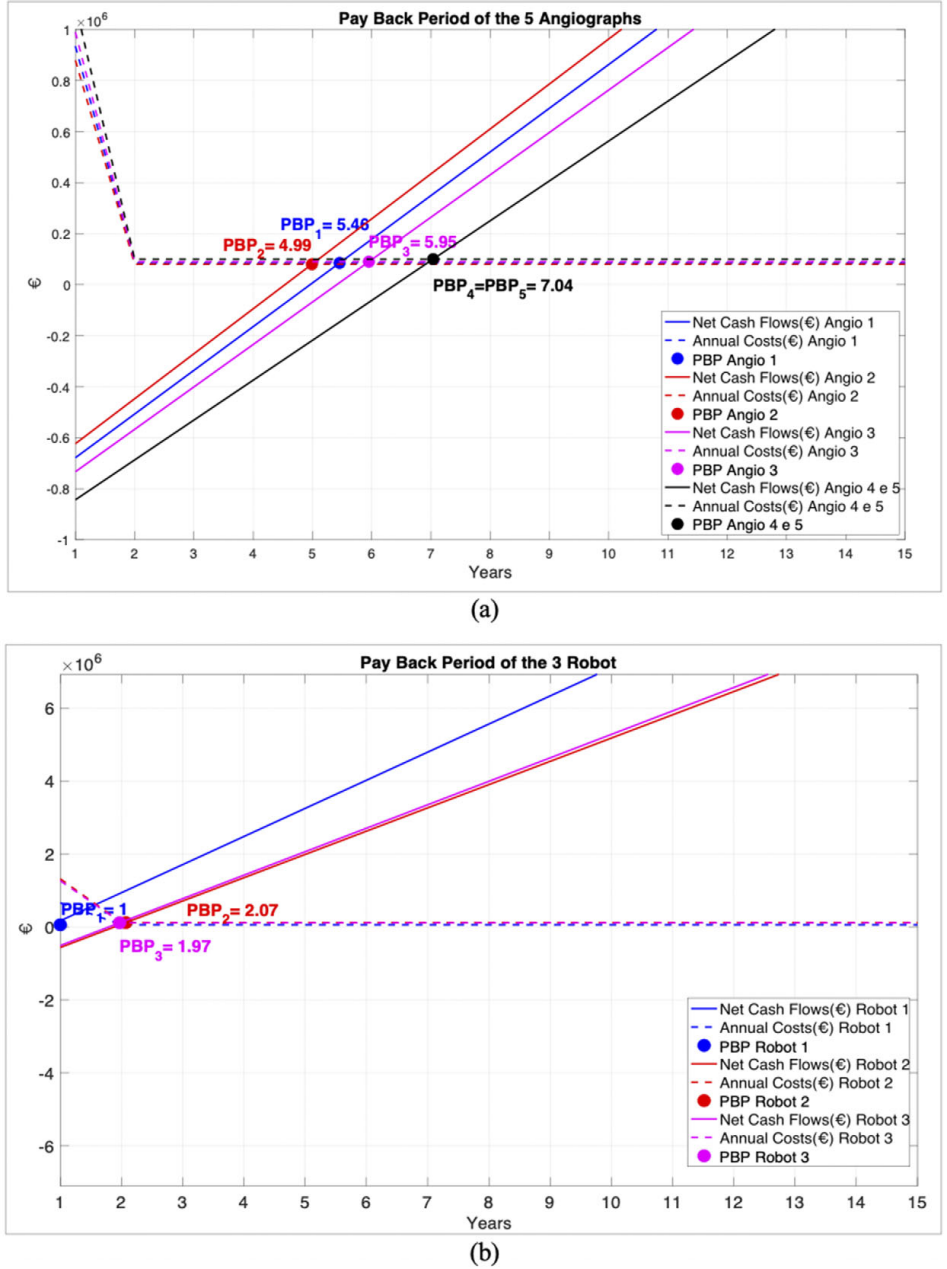
Representation of the Payback Period index, consisting of the number of years required for the annual cash flows generated by the investment over time to allow the Hospital Trust to recover the initial expenditure incurred to introduce the new technology. (A) Payback period of angiographs. (B) Payback period of neurosurgery robots.

Angiograph 4 is among the safest options but slightly lags in other domains. Angiograph 5 excelled in clinical effectiveness (29 percent) but had a 7-year PBP. PBP analysis, represented in Figure 2A, assigned the highest score to Angiograph 2 with a PBP equal to 5 years.

Economic analysis considered DRGs divided between neurosurgical and vascular surgery procedures. Break-even analysis (Figure 1F) showed minimum required procedures: 185 performances for Angiograph 1, 174 for Angiograph 2, 195 for Angiograph 3, and 217 performances for both Angiograph 4 and Angiograph 5.

Estimated PBP for both robots and angiographic is shorter than their 8-year lifespan: 1–2 years for robots and 5–7 years for angiographic systems. To evaluate the quantities needed for quicker return on investment, a 3-year timeframe was set for angiographs and 1-year for robots due to their already short payback periods.

**Figure 3.**
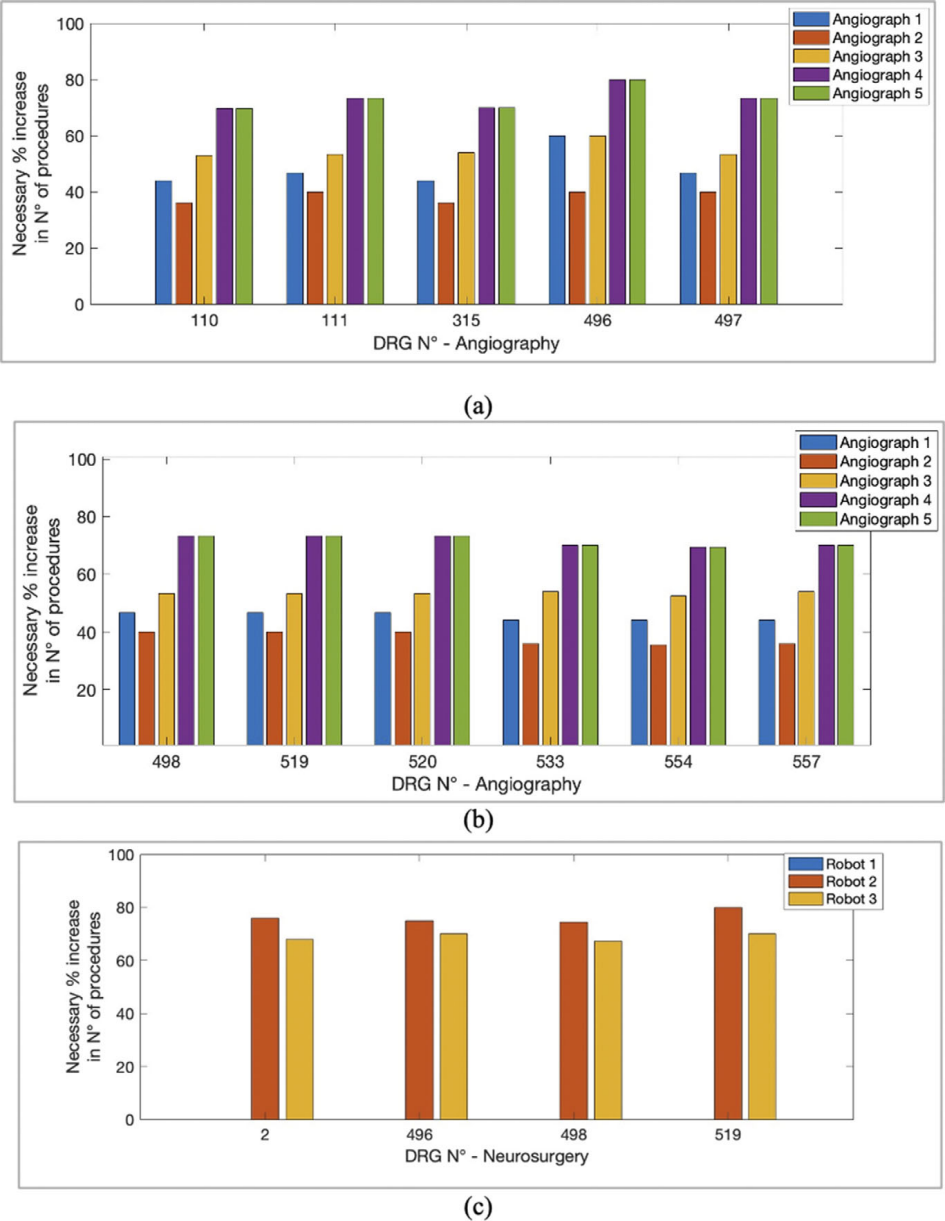
Predictive analysis considering a payback period of a specifical number of years for the examined technologies. Bar graphs showing the percentage of extra procedures, compared with the annual amount already executed by the Hospital for each DRG, to be performed to repay the investment within the set years for each DRG. (A), (B) Percentage of extra procedures for all angiography devices for each angiography-related DRG assuming a payback period of 3 years for all angiographs. (C) Percentage of extra procedures for all neurosurgery robots for each neurosurgery-related DRG assuming a 1-year return to cover the initial investment.

BEP and PBP results were used as input in the hierarchical model, followed by a predictive analysis assuming a payback period of 3 years for all angiographs. This analysis provided the new quantities of services to be delivered for each DRG associated with the five angiographs (Figure 3A,B).

Predictive analysis for robots aimed to minimize the timeframe, assuming a 1-year return to cover the initial investment. Figure 3C shows the results for neurosurgical DRGs, where the absence of the first robot’s bar system means that its choice would not improve the annual procedures required to cover the investment.

**Figure 4.**
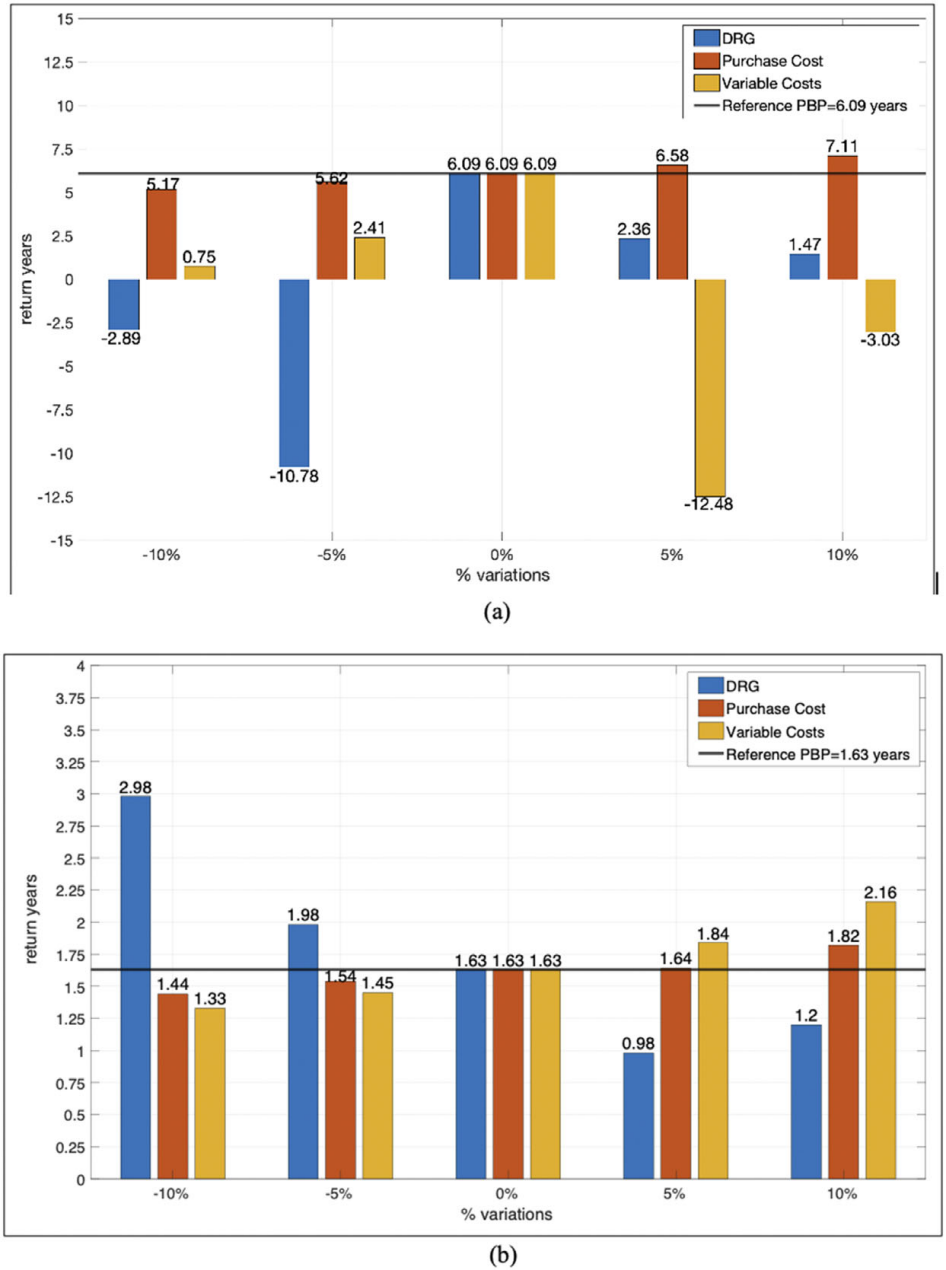
Indicative evaluation of significant parameters in the variation of the Payback period. Percentage variations of the DRG, variable costs, and fixed costs have been applied to both the angiography devices (Figure 4(A)) and the robots (Figure 4(B)).

Sensitivity analysis (Figure 4) showed that changes in DRG values and variable costs had the most significant impact on the PBP, while fixed costs had a more limited effect. Bar graphs illustrate the results for both angiographs and robots. The average values were reported for the five angiographs and the three robots since disparities between the different devices, belonging to the same category, were minimal. Two key parameters, DRG and variable costs, affect payback years differently: increasing DRG value decreases payback years, while rising variable costs reduces them. For angiographs, payback years can become negative with moderate cost increases (5 percent and 10 percent) due to the annual contribution margin (revenue minus costs) that heavily influences depreciation. Purchase cost, affecting fixed annual costs, had limited impact on payback years. The analysis identified intervention parameters to reduce or increase the depreciation period. No robot showed negative payback years, and the highest payback years were equal to 2.98, remaining compatible with the equipment’s useful life. This disparity between robots and angiography devices is due to the differences in the cost’s magnitude.

## Discussions

The study focuses on optimizing the evaluation process through an HTA toolbox designed to encompass the multidisciplinary aspects necessary for assessing biomedical equipment. Its theoretical contribution fits into broader discussions: first, the necessity of an objective and standardized decision-making process in healthcare (17). Second, the necessity of improving MCDA methods to support HTA agencies in setting healthcare priorities (14). This tool facilitates the selection of parameters, their scoring, and monitoring of all crucial factors when choosing between competing devices. The introduction of an algorithm into the toolbox was crucial in translating qualitative assessments into quantitative outcomes. Theoretically, the toolbox improves HB-HTA by integrating AHP-based evaluation with economic modeling; practically, it supports hospitals in efficiently optimizing resource allocation and investment planning.

An AHP-based algorithm offers a systematic and standardized method for HTA evaluations. The toolbox’s economic analysis feature offers both short- and long-term analysis options, providing two key economic indicators: the BEP to avoid economic loss and the PBP for recovering purchase expenses. This balances clinical needs and economic sustainability, especially when DRG tariffs do not fully cover service costs. The analysis provided by the toolbox allows for careful handling of the resources, crucial for long-term economic sustainability of the adoption of healthcare technology.

The economic analysis within the toolbox enables also a predictive analysis by allowing the setting of a payback period without limiting its simple computation, which is typically performed with the PBP method. It identifies the new volumes required for each DRG to repay the expenditure incurred on technology within the set timeframe. This dual approach bridges the gap between financially advantageous but not always economically beneficial investments.

A further analysis highlighted two crucial parameters: DRG value and variable costs, which have opposite effects on payback years. Increasing the DRG value decreases payback years, while increasing variable costs raises them. This involves carefully examining the costs (variable costs) and revenues (DRGs) associated with the use of the equipment, with the aim of generating positive cash flows. Thus, PBP and DRG indicator could help the hospitals’ HTA team plan and handle future strategic investments to build a sustainable healthcare system made of clinically efficient technologies.

At San Giovanni Addolorata Hospital, this study marked the first implementation of an automated AHP procedure. Previously, the evaluation process relied on manual assessments and non-standardized methods based on questionnaires and spreadsheet tools. This automation significantly reduced evaluation time and minimized human errors, facilitating a more efficient, consistent, and reliable decision-making process.

However, the quantitative approach supported by the toolbox allowed for a detailed evaluation of the equipment’s technical performance, highlighting the clinical benefits derived from adopting advanced technologies for safe procedures. Furthermore, it facilitated more effective monitoring and management of costs associated with introducing new technologies, which are crucial for the hospital’s financial equilibrium. By providing accurate projections and feasible recoupment times, the toolbox addressed the previous tendency of the HTA team to overestimate cost recovery periods. Moreover, it strengthens methodological consistency in HB-HTA, combining structured evaluation with economic modeling in a reproducible framework.

Although it was created within an Italian hospital, the toolbox is compliant with national and regional regulations and EUnetHTA principles and can be adopted by other hospitals. Its modularity and user-friendly MATLAB-based interface makes it easy to adapt to local priorities and operational needs. Further developments like integration within interoperable platforms and language localization will facilitate adoption at the European level even further.

## Limitations

Limitations include reliance on only Saaty’s scale for the AHP, whereas other existing scales may provide alternatives for assigning weights and evaluating criteria (27). The adopted rapid HTA approach currently excludes social and organizational domains, unlike full HTA reports. Additionally, the toolbox remains operator-dependent, and this factor could compromise the objectivity of the system. In future, the interface could also integrate other MCDA methods (28;29), expanding its flexibility to support different decision-making scenarios. The toolbox is also designed to be used when evidence is limited, thanks to its flexible structure; however, expert input from experienced academic and hospital-based teams remains essential to ensure appropriate and reliable use. Eventually, the scope of the evaluation was limited to two technologies. However, these differ in purpose and complexity, and the toolbox is designed to be adaptable to various healthcare technologies.

## Conclusions

The proposed toolbox aims to support the HTA team in evaluating healthcare technologies. It serves as a mediating element among clinical and economic needs of the HTA team, uncertainties in choosing between market technologies, and the requirement for a standardized, semi-automatic, and multi-parametric assessment model. This work aims to improve coherence and multidisciplinarity of healthcare decisions through a semi-automated strategy, aiming to achieve economic equilibrium and improve hospital’s internal operations by increasing the number of annual services provided.

The study highlights the user-friendly nature of the toolbox and its applicability across various HTA contexts, allowing adaptation to other hospitals by customizing sub-criteria and integrating regional economic data, such as region-specific DRG tariffs. Furthermore, provides hospitals with a more systematic, short- and long-term assessment. Future integration of deep learning models for data analysis, weighting criteria and identifying priority areas based on past data could support more data-driven decisions.

In conclusion, adopting the HTA toolbox at San Giovanni Addolorata Hospital represents a significant step toward more informed and sustainable clinical and managerial decisions. The multidisciplinary approach, process automation, and in-depth analysis of costs and benefits are crucial elements for ensuring superior quality care and efficient management of HTA team’s resources in the long term.

## Supporting information

Bini et al. supplementary material Bini et al. supplementary material

## Supplementary material

The supplementary material for this article can be found at http://doi.org/10.1017/S0266462325103176.
