# Supplementary material for: An HTA systems decision-support toolbox for short and long-term healthcare and economic perspectives in an Italian hospital
Source: Int J Technol Assess Health Care. 2025 Oct 16;41(1):e77. doi: 10.1017/S0266462325103176 (PMC12592966; doi:10.1017/S0266462325103176)

**Appendix A**

$${BEP}_{corporate}=\frac{CFT}{p_{thoughtful}-c{vu}_{thoughtful}}$$

Formula S1: Formula for calculating the first economic indicator within the toolbox's economic analysis is as follows. The corporate BEP represents the minimum total quantity of all services deliverable with the technology under review, weighted by the actual annual quantities delivered. CFT comprises the annual fixed costs attributable to the use and maintenance of the technology. *P_thoughtful* is the weighted revenue source, and *cvu_thoughtful* is the unit variable cost associated with the use of the technology, weighted by the services deliverable.

$$p_{thoughtful}=\sum_{i=1}^{n} p_{i}*\frac{q_{i}}{q_{tot}}$$

Formula S2: Formula for calculating the weighted price *P_thoughtful* where n represents the Diagnosis-Related Groups (DRGs) related to the services deliverable with the technology under consideration; *q_i/q_tot* corresponds to the volume mix relative to the i-th service associated with one of the included DRGs, relative to the total quantity of services delivered annually across all considered DRGs.

$${cvu}_{thoughtful}=\sum_{i=1}^{n} {cvu}_{i}*\frac{q_{i}}{q_{tot}}$$

Formula S3: Formula for calculating the weighted unit variable cost *cvu_thoughtful* where n represents the Diagnosis-Related Groups (DRGs) related to the services deliverable with the technology under consideration; *q_i/q_tot* corresponds to the volume mix relative to the i-th service associated with one of the included DRGs, relative to the total quantity of services delivered annually across all considered DRGs.

$$E=\sum_{t=1}^{k} F_{t}$$

Formula S4: Implementation formula of the Payback Period. *E* indicates the initial outlay; *k* indicates the years required to recover the initial investment cost; *Ft* indicates the annual net cash flow generated by the investment over time, calculated as the difference between total revenues (*RT*) and total costs, comprising the sum of total variable costs (*CVT*) and fixed costs (*CF*).

$$Q=\frac{AC+nCF}{n(mdcu)}$$

Formula S5: Formula for computing *Q*, the total amount of breakeven funds to be disbursed to recover the investment within the desired payback years; *n* - desired payback years; *CF* - fixed costs; *AC* - purchase cost corresponding to the initial expenditure borne by the hospital; *mdcu-unit* contribution margin.

**Appendix B**
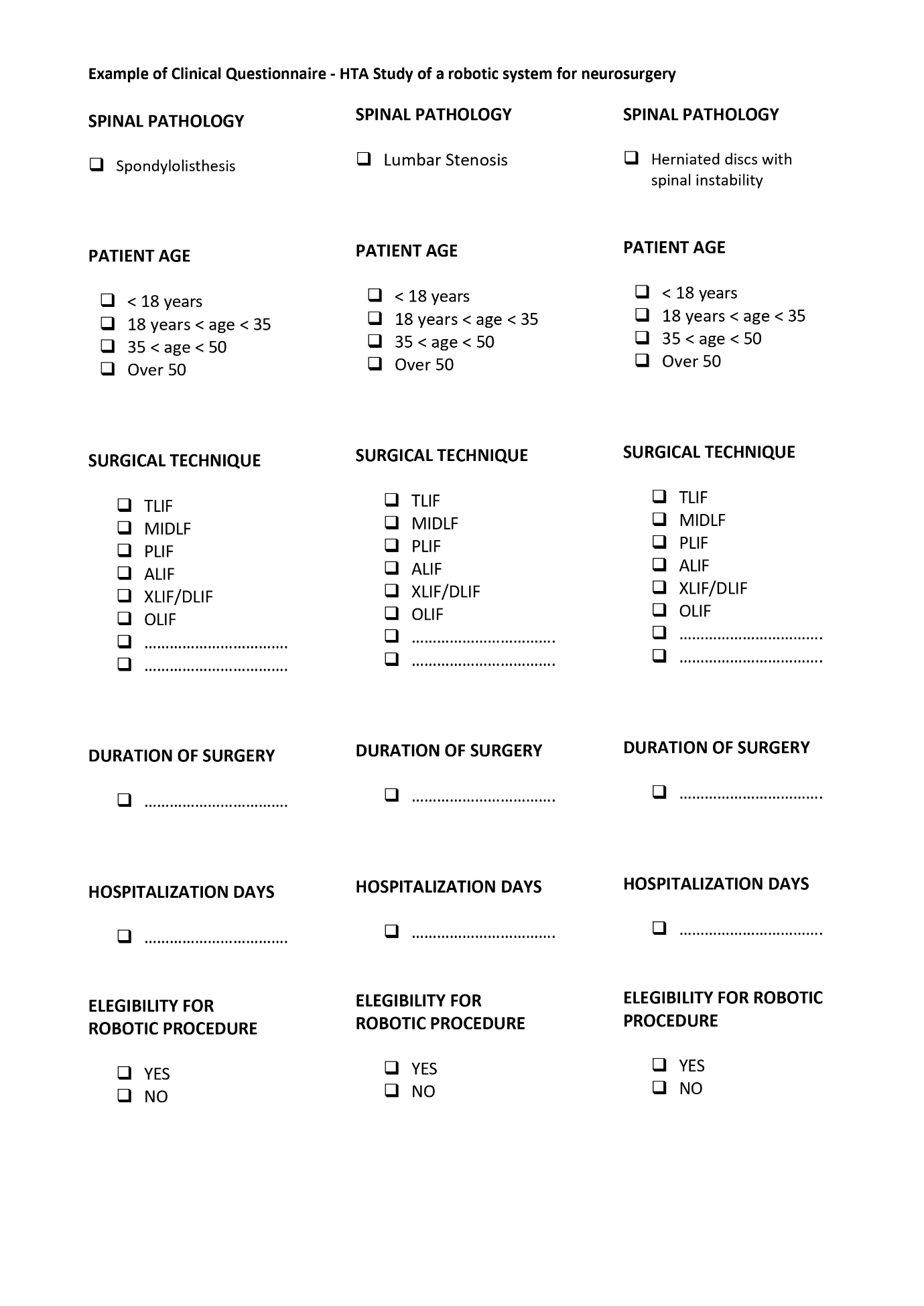

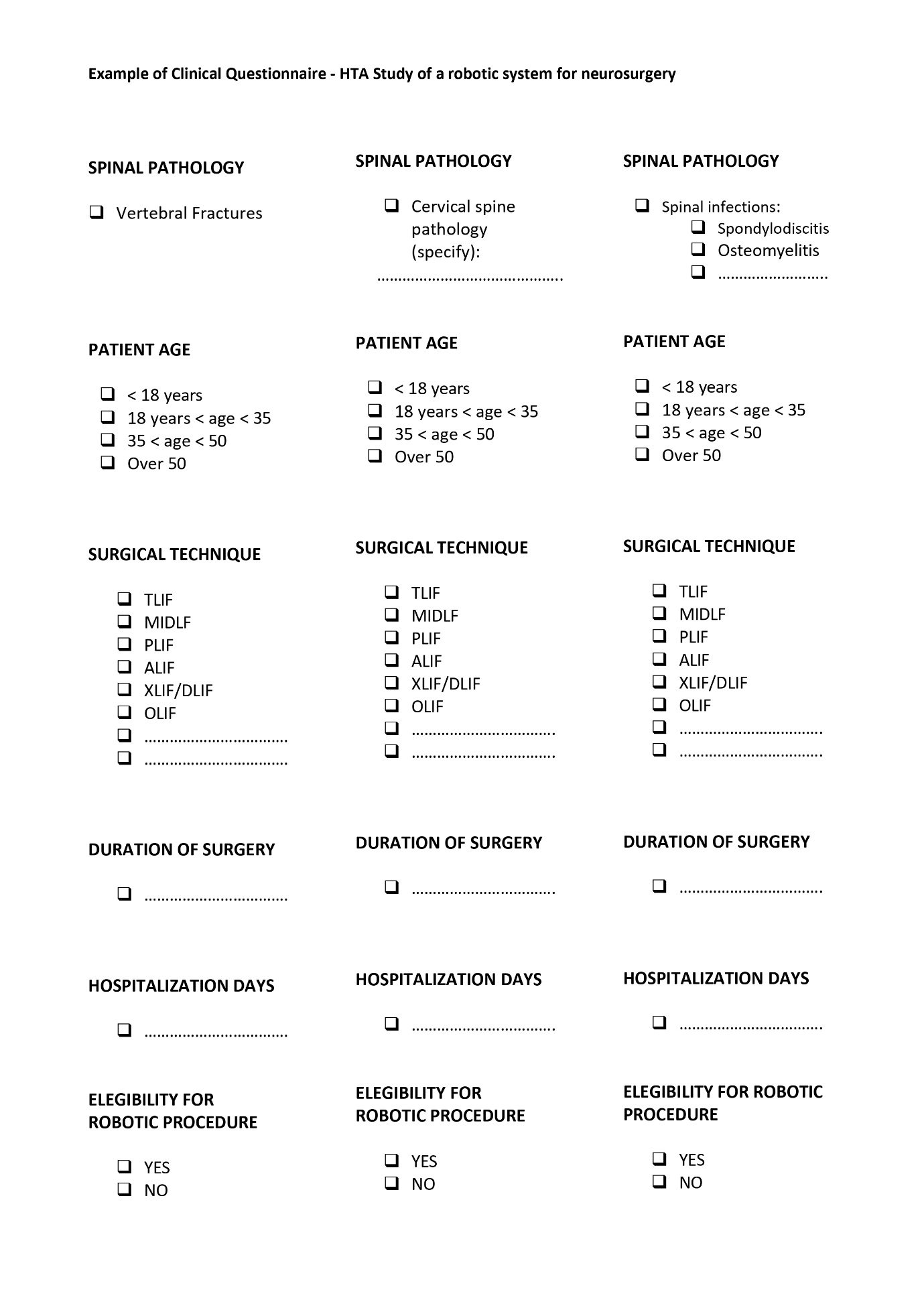

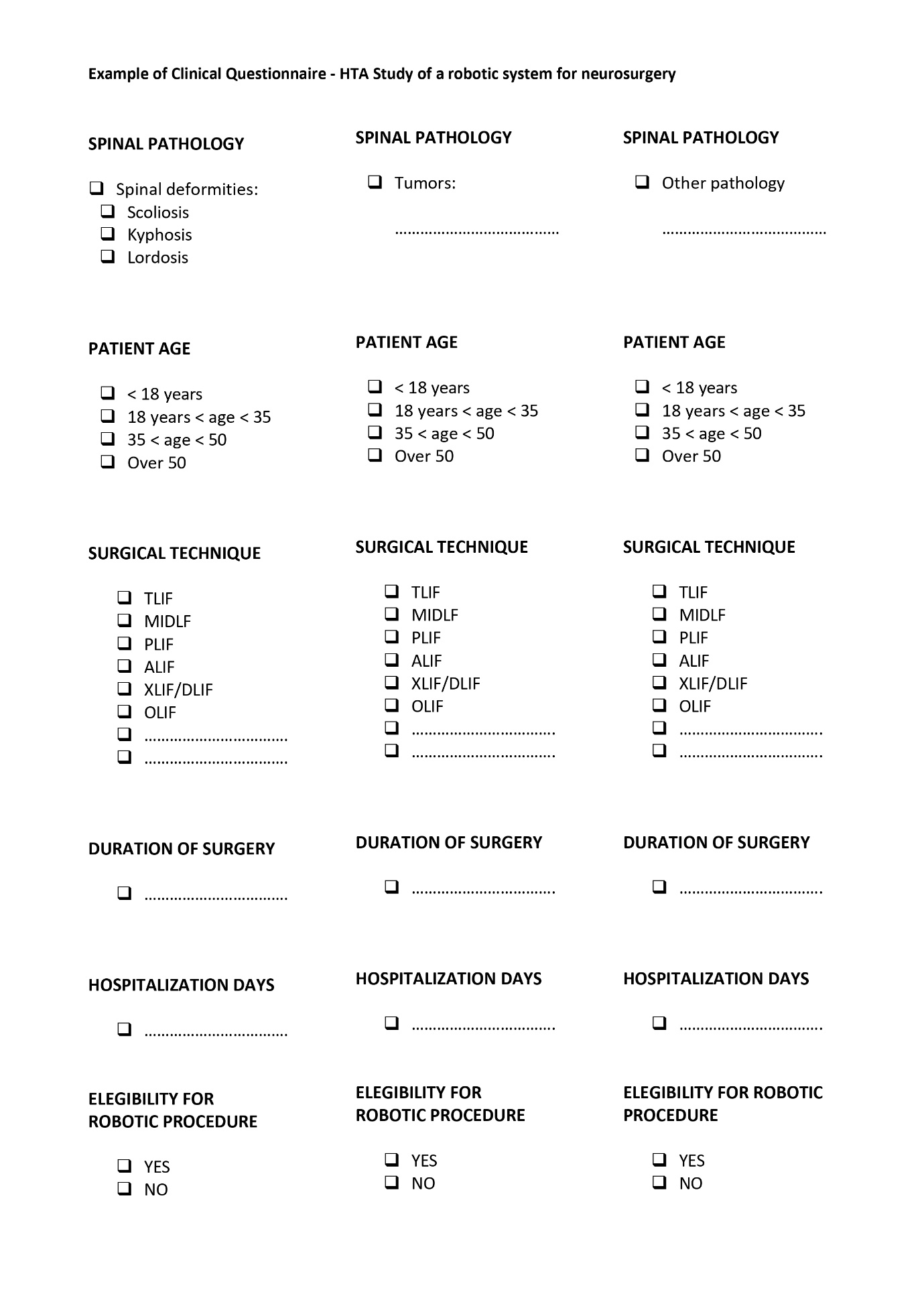

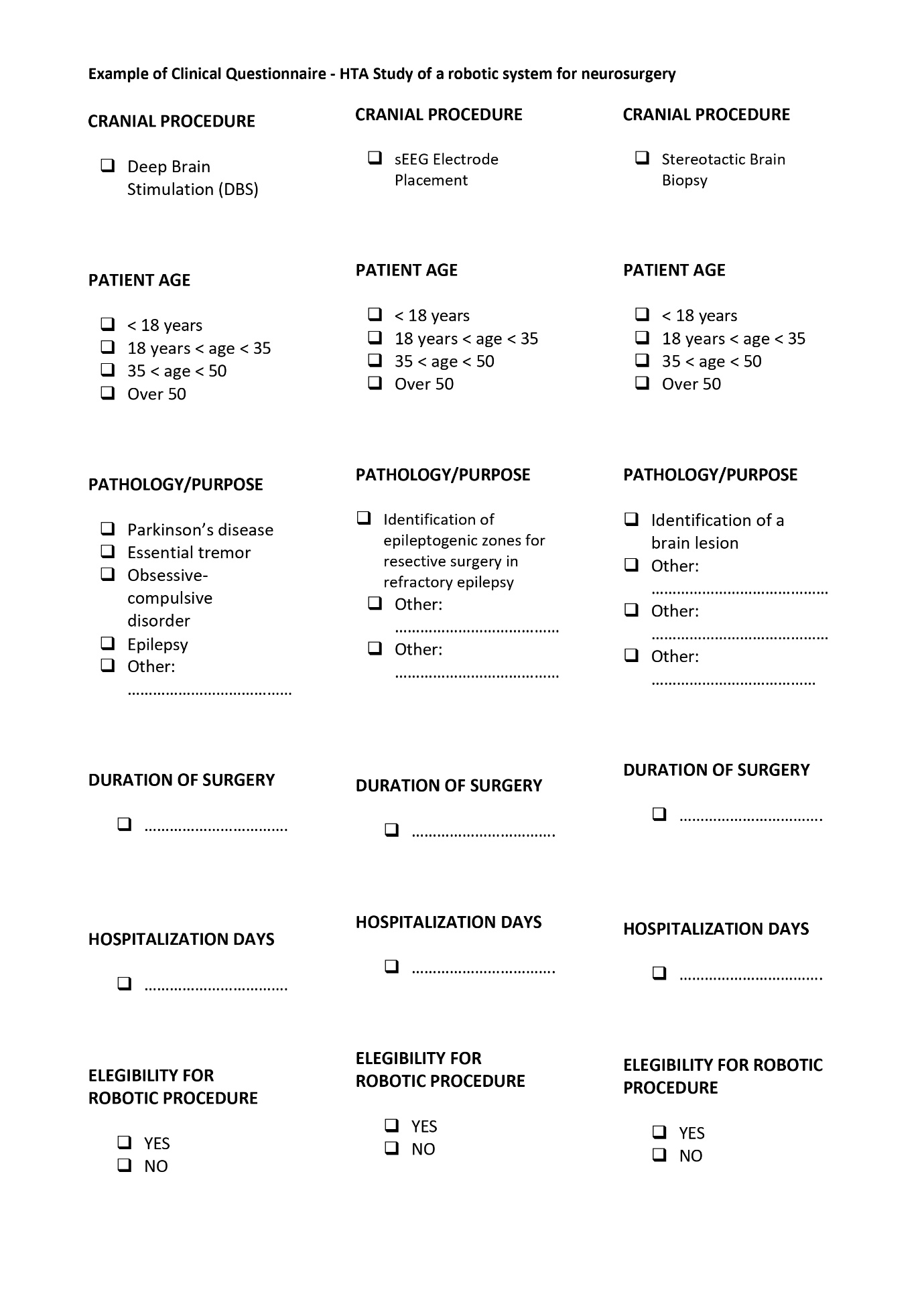

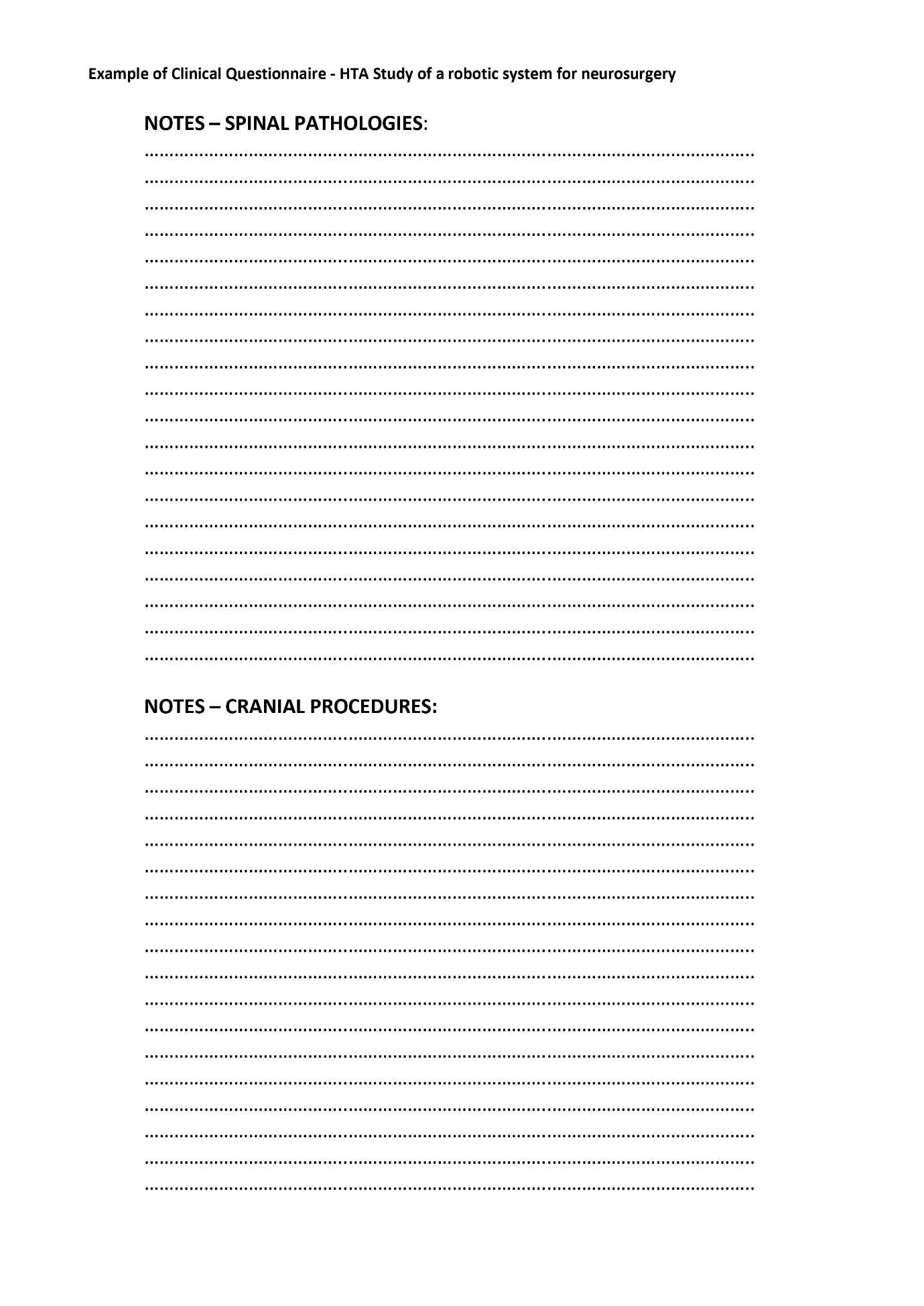

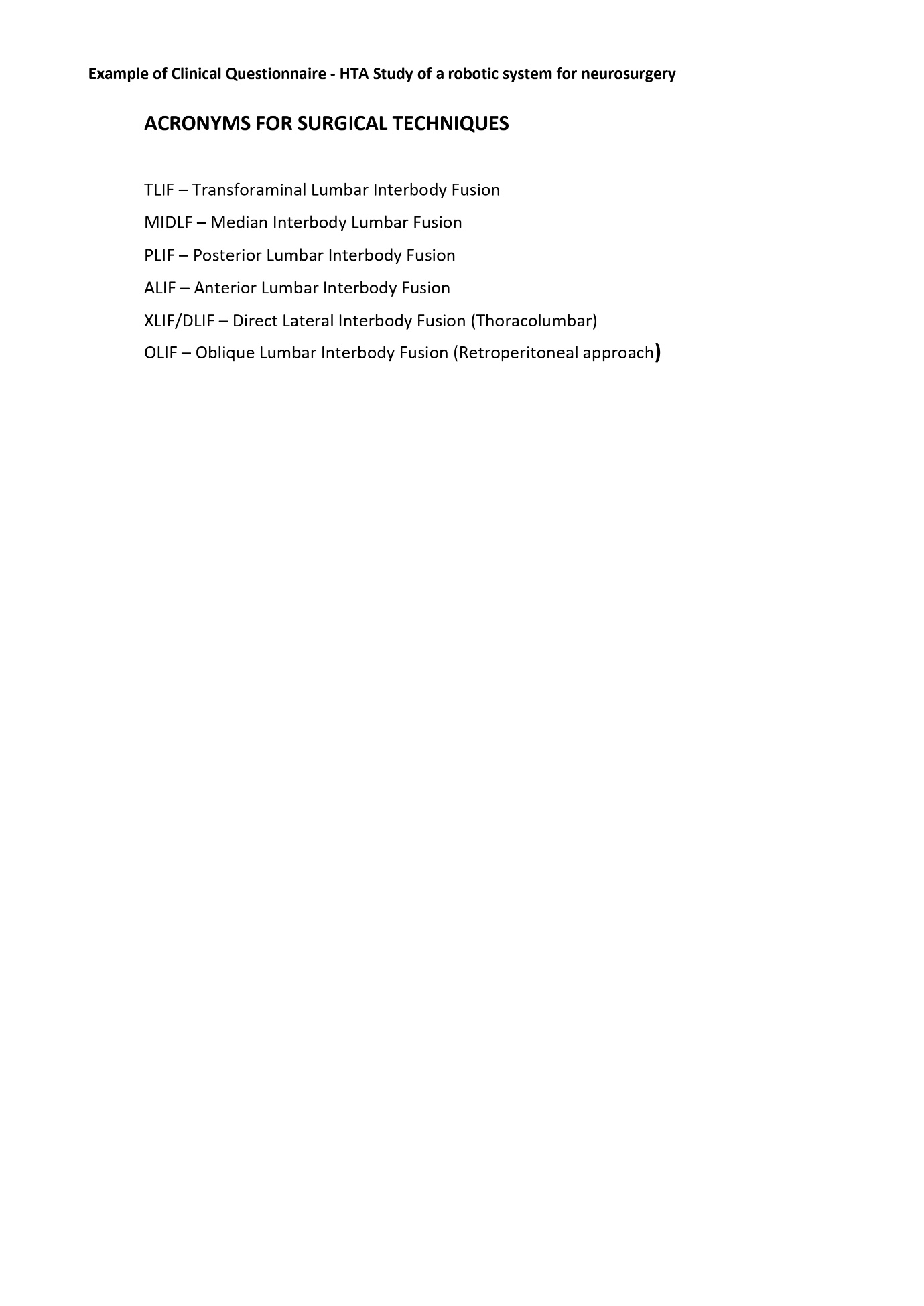


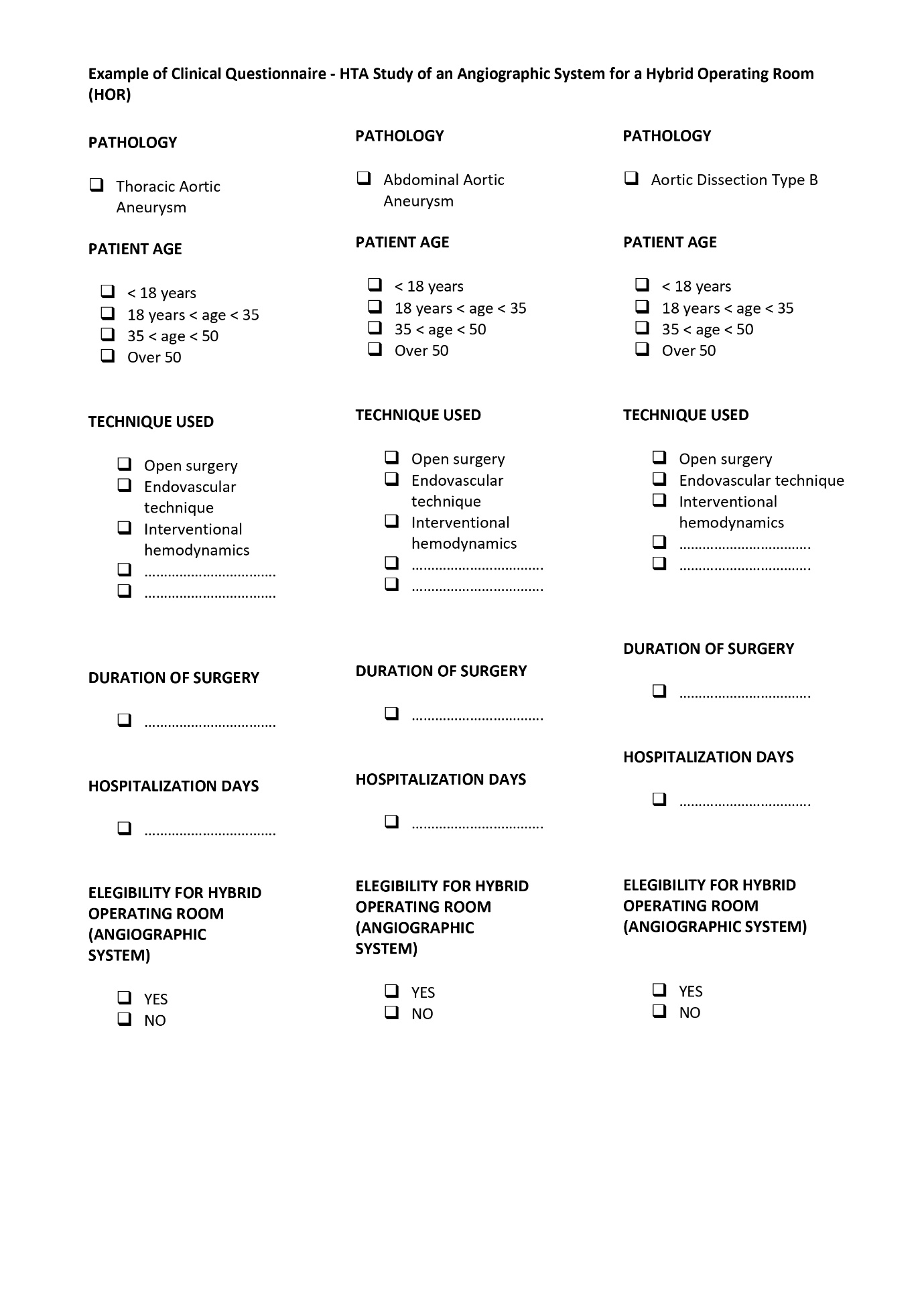

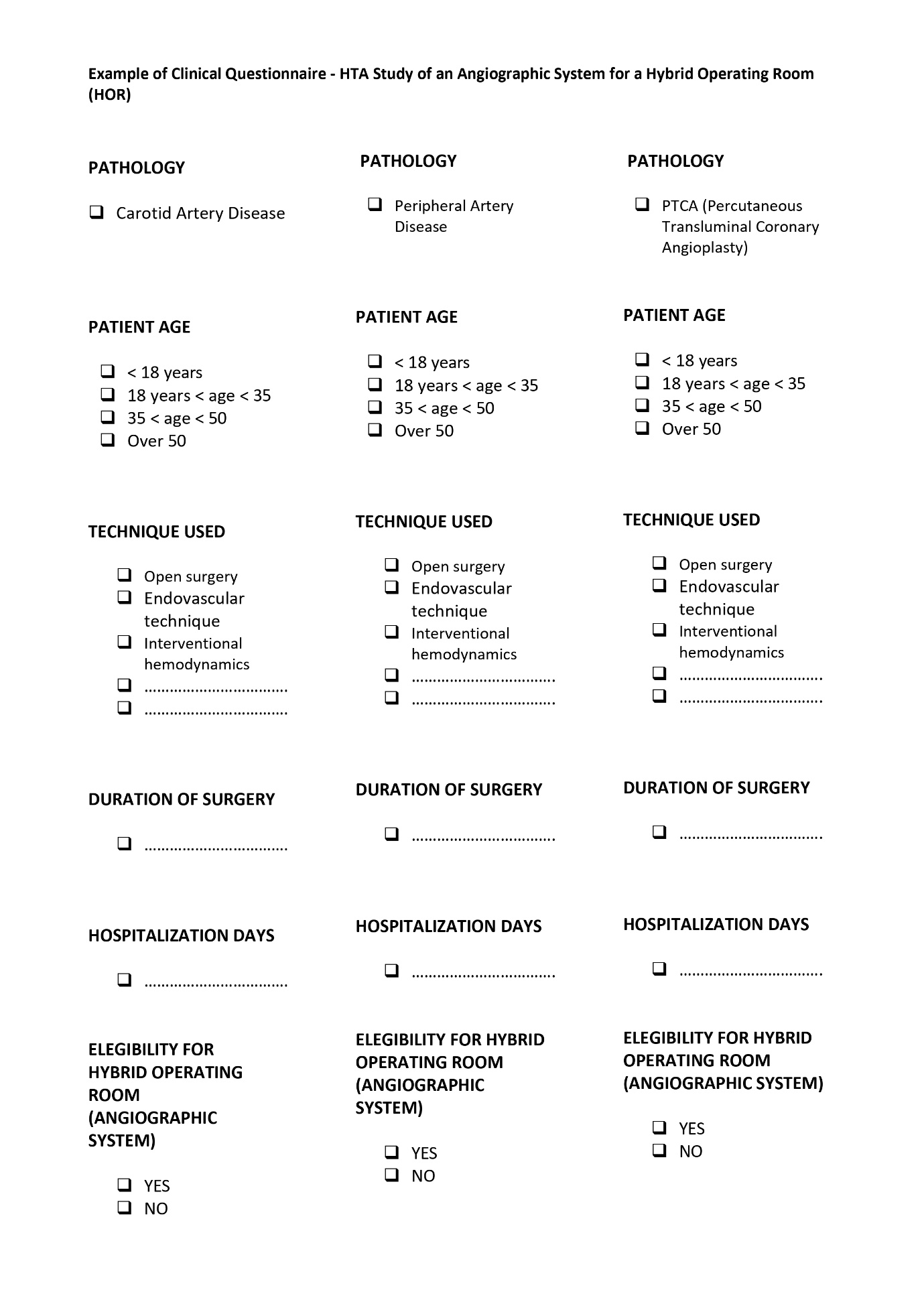

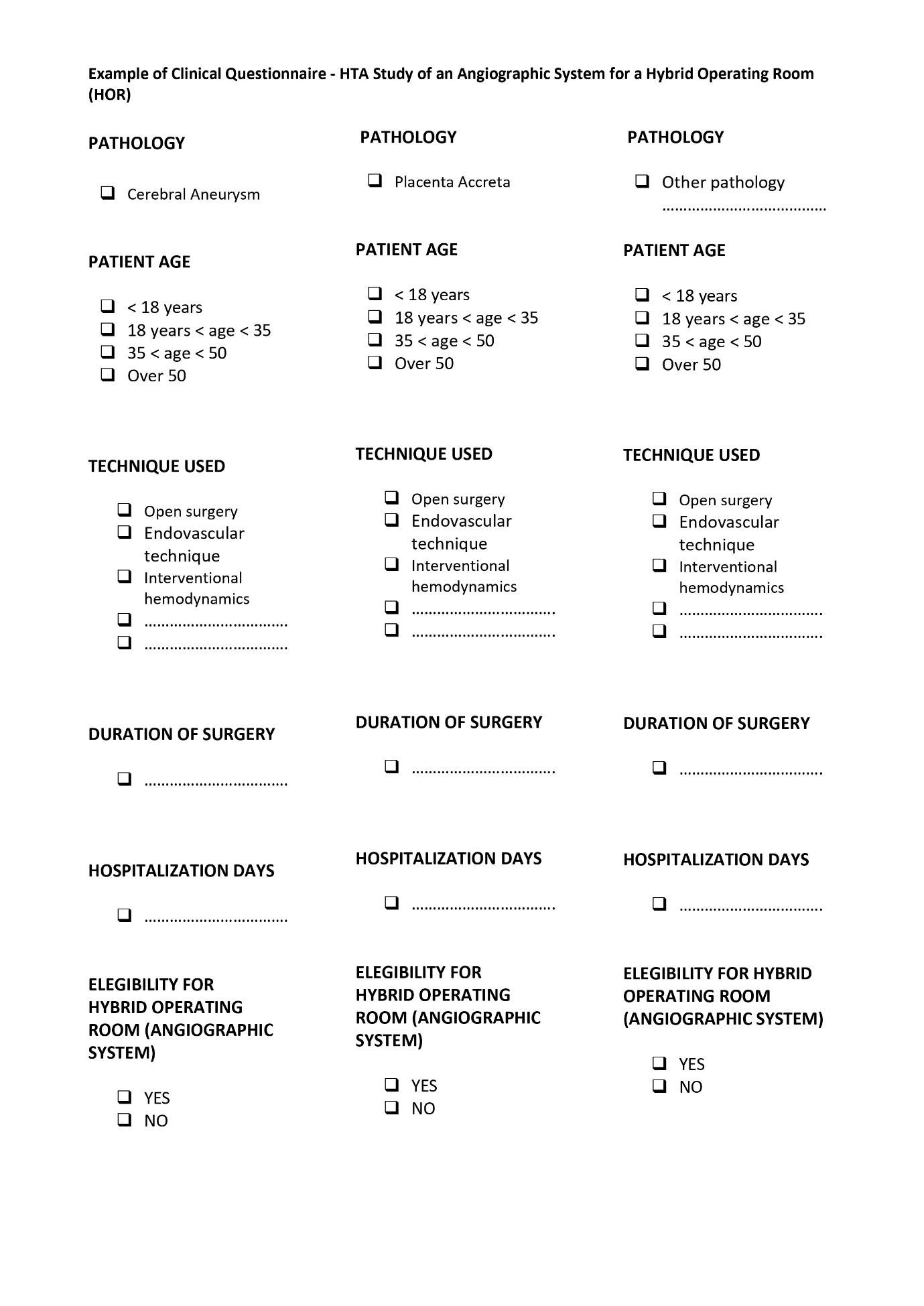

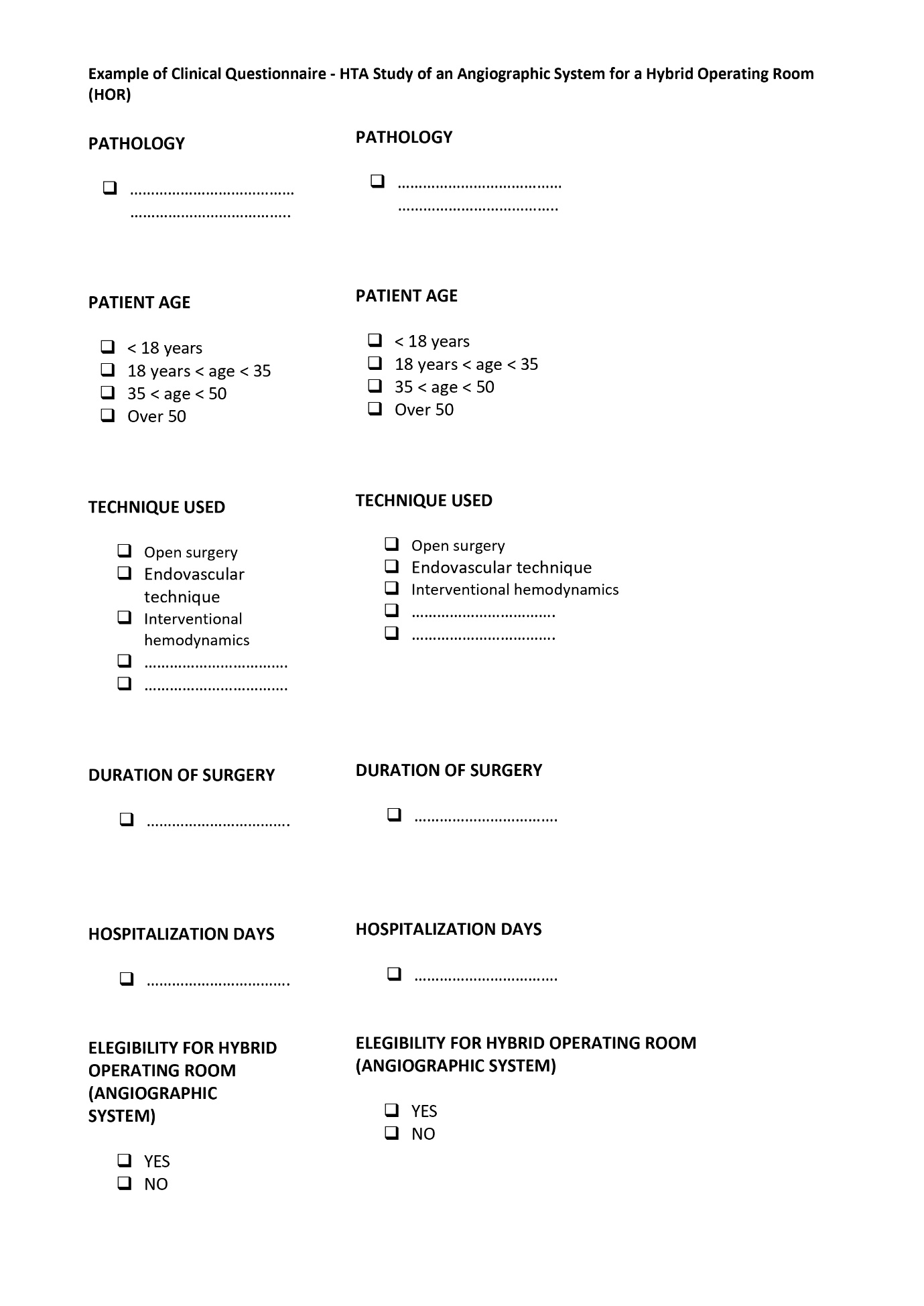

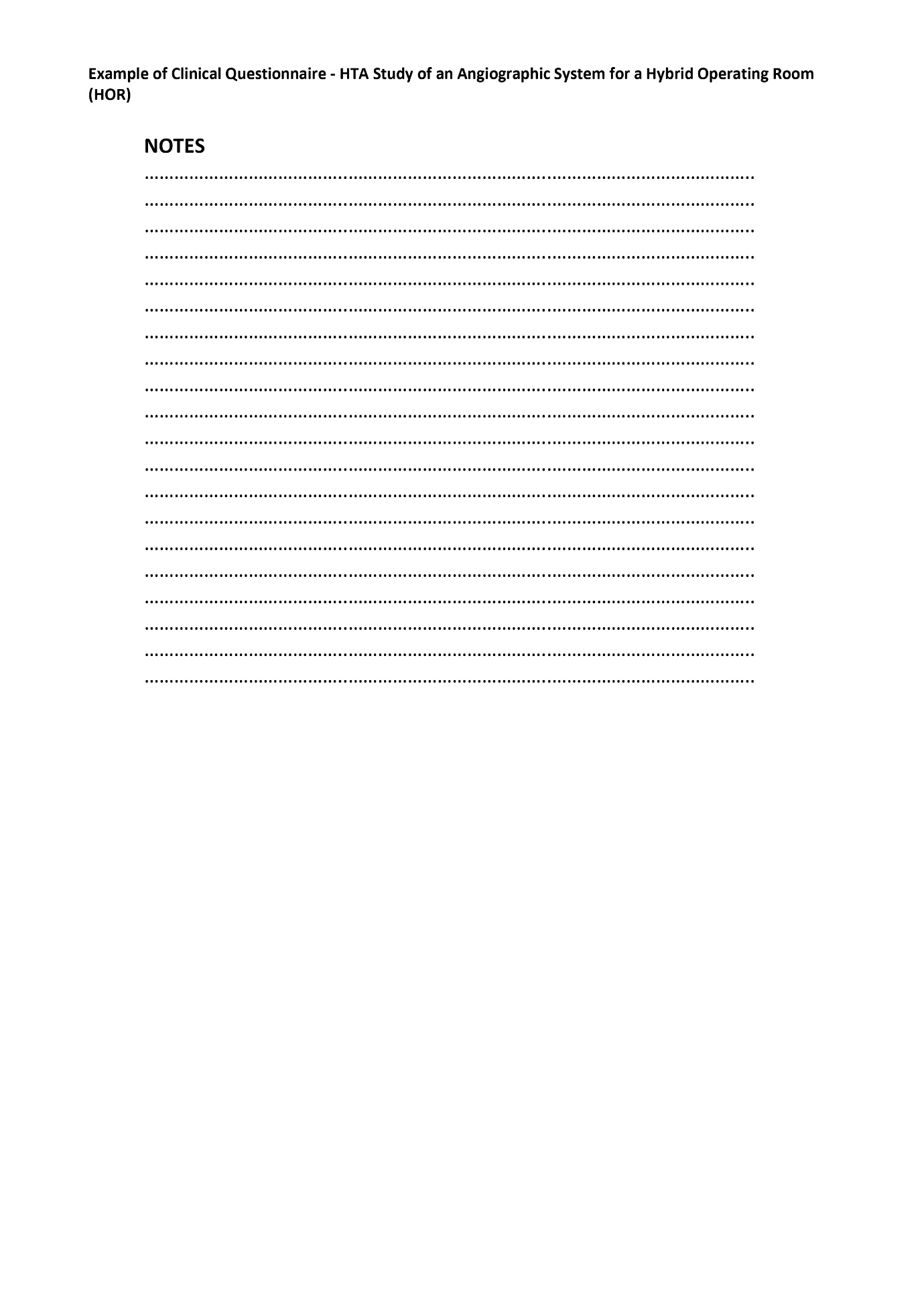

Supplement: Bini et al. supplementary material [file S0266462325103176sup001.docx]
